# Reducing the Cost of Laparoscopy: Reusable versus Disposable Laparoscopic Instruments

**DOI:** 10.1155/2014/408171

**Published:** 2014-07-22

**Authors:** Dimitrios K. Manatakis, Nikolaos Georgopoulos

**Affiliations:** 2nd Surgical Department, Athens Naval and Veterans Hospital, 70 Deinokratous Street, 11521 Athens, Greece

## Abstract

Cost-effectiveness in health care management is critical. The situation in debt-stricken Greece is further aggravated by the financial crisis and constant National Health System expense cut-downs. In an effort to minimize the cost of laparoscopy, our department introduced reusable laparoscopic instruments in December 2011. The aim of this study was to assess potential cost reduction of laparoscopic operations in the field of general surgery. Hospital records, invoice lists, and operative notes between January 2012 and December 2013, were retrospectively reviewed and data were collected on laparoscopic procedures, instrument failures, and replacement needs. Initial acquisition cost of 5 basic instrument sets was €21,422. Over the following 24 months, they were used in 623 operations, with a total maintenance cost of €11,487. Based on an average retail price of €490 per set, projected cost with disposable instruments would amount to €305,270, creating savings of €272,361 over the two-year period under study. Despite the seemingly high purchase price, each set amortized its acquisition cost after only 9 procedures and instrument cost depreciated to less than €55 per case. Disposable instruments cost 9 times more than reusable ones, and their high price would almost equal the total hospital reimbursement by social security funds for many common laparoscopic procedures.

## 1. Introduction

The wide adoption of the concept of minimally invasive laparoendoscopic surgery and the concomitant progress in technology have reduced the prices of sophisticated laparoscopic instruments; however, cost of laparoscopy remains generally higher than that of open surgery. In most clinical studies, higher operating theatre cost is partly offset by shorter hospital stays, less medication requirements, shorter periods of convalescence, and faster return to work and normal activity [1–6]. On the other hand, multiple cost studies, already from the 1990s, have shown that reusable laparoscopic instruments (RLIs) are a valuable asset for surgical departments in terms of cost reduction [7–15].

In an effort to minimize the cost of laparoscopic surgery, without compromising quality of health services, our department introduced RLIs in December 2011, under the pressure of budget limitation. The primary aim of this study was to assess potential cost reduction of laparoscopic procedures performed between January 2012 and December 2013, in the field of general surgery. The secondary aim was to calculate annual maintenance expenses of RLIs and investigate possible ways of further cost limiting.

## 2. Materials and Methods

Hospital records, invoice lists, and operative notes between January 2012 and December 2013 were retrospectively reviewed and data were collected on type and number of laparoscopic operations, instrument failures, and replacement requirements. Maintenance cost was calculated as the sum of resterilization, repackaging, repair, and replacement expenses. For comparison purposes, retail prices of disposable laparoscopic instruments (DLIs) were obtained from the National Price Observatory for Health Supplies. Disposal cost for DLIs, based on weight of waste, was practically negligible.

Cost of insufflation and suction-irrigation tubes, clip appliers, retrieval bags, mesh patches, staplers, and so forth, was not included in the analysis, since there is no choice between reusable and disposable forms.

## 3. Results

In December 2011, 5 basic sets of RLIs were purchased (AESCULAP, B-BRAUN, Tuttlingen, Germany), with initial acquisition cost of €21,422. Each set consists of a curved monopolar dissector, two atraumatic grasp monopolar forceps with ratchet, a pair of Metzenbaum monopolar scissors, a suction-irrigation device, a Veress needle, a Hasson cannula, and two 12 mm and two 5 mm bladeless trocars with replaceable rubber valves. Over the following 24 months, they were used in 623 laparoscopic operations (Table 1), covering a wide range of upper and lower GI tract pathologies and obesity.

Two-year maintenance cost amounted to €11,487. Resterilization and repackaging of each set were calculated at €5 per procedure, costing €3,115 for the two-year period under study, while replacement and repair costs came to €8,372, with requirements limited to torn trocar valves and a broken grasper forceps (Table 2). Trocar valves were replaced after an average of 10 procedures, while scissors were sent for sharpening after 60–80 operations.

Based on an average retail price of €490 per set, estimated cost of 623 procedures with DLIs would come to €305,270. The actual total instrument cost was €32,909, creating savings of €272,361 over two years.

## 4. Discussion

Cost-effectiveness in health care management has always been a major issue. The situation in debt-stricken Greece is further aggravated by the financial crisis and constant National Health System expense cut-downs. Hospital administrations are called to drastically reduce their expenditure and use their limited resources wisely.

Our preliminary study of reusable versus disposable laparoscopic instruments, based on a projected number of 200 laparoscopic operations per annum, predicted net yearly savings of around €80,000. Two years later, the results of this analysis clearly confirm our expectations. Despite the seemingly high purchase price, each set amortized its acquisition cost after only 9 laparoscopic operations. Initial cost was 65% of the total two-year expenditure and depreciated to less than €55 per patient. This figure is expected to fall even more, with every future use of the instruments.

As per annual maintenance cost, roughly 30% covered the relatively inflexible expenses of resterilization and repackaging, while 70% pertained to replacements and repairs. Worn-out valves need replacement more often than any other instrument part [10, 12]. This results in frequent air leaks and higher CO_2_ volume to maintain the pneumoperitoneum at the desired pressure. We have observed that most of the leaking valves were torn at the rubber seal, a result of forceful insertion/withdrawal of instruments.

The cost analysis revealed more useful conclusions. Laparoscopic instrument cost would be 9 times higher with disposable compared with reusable instruments. More importantly though disposable instrument cost alone would cover almost half of the total hospital reimbursement by social security funds for many common laparoscopic procedures (Table 3) [16].

Further reduction in cost will chiefly result from increasing the number of uses, reflecting the decreasing depreciation cost per case. Expanding use of RLIs in the fields of urology and gynecology would add another 50 to 100 procedures annually. On the other hand, maintenance expenses, at €5,750 per year, are already quite acceptable and rather fixed.

Much discussion has occurred in the past regarding the advantages and disadvantages of reusable versus disposable instruments [17]. The rationale behind initial skepticism against RLIs included not only high acquisition and maintenance cost, but also issues of possible transmission of infectious disease to patients and medical personnel, instrument inefficiency, and lack of durability [18, 19]. However most of these studies date back to the 1990s. With technological progress, newer designs, and appropriate training; these problems, if once existing, are virtually obsolete.

Despite speculative arguments, sterilization proved to be adequate, when manufacturer's instructions are followed, and we could not find any case report in the literature of infectious disease transmission through inadequately processed RLIs [7, 9, 20]. The only difference that we detected was that mechanized cleaning systems are reported to be more efficient and safer than manual washing [21]. The cheap alternative of resterilizing single-use instruments may be feasible but is generally not advised [9, 22–25]. These instruments are not designed to be disassembled and dead spaces inside the instrument may harbor residual debris, which are difficult to remove between procedures. This makes the surgeon ultimately responsible for any mishap, raising ethical and juridical concerns.

Modular configuration, adopted by most manufacturers, permits dismantling of the instruments, while maintaining a simple yet robust design. Thus repairs are facilitated, since only the faulty part can be replaced, with minimal cost. Moreover, access is allowed throughout the shaft and the handle, allowing for optimal cleaning and effective sterilization.

Skeptics also voice that ergonomics and efficacy of DLIs are unequaled, since they are “new” every time and this may indeed be true up to a point. Reusable instruments are more prone to technical issues but should be considered an investment for every surgical department and with careful handling and maintenance, the normal wear and tear can be kept to a minimum [15]. Other than a broken grasper working tip, due to an accidental fall to the ground, we had no major instrument failures. A hybrid model of reusable instruments backed up by a small number of disposable ones, for immediate availability in cases of technical problems, appears as the optimal solution, balancing cost-effectiveness with reliability [10].

Next to trocar valves that required replacement every 10 procedures, scissors needed sharpening after 60–80 operations, whereas bladeless trocars do not require sharpening at all. Reusable trocars possibly require more force during insertion and this could be associated with more intra-abdominal injuries [26]. Our team has not observed any visceral or vascular injuries with either the Veress or the Hasson approach. Irrespectively of trocar design, it is imperative always to insert the trocars under direct vision, to minimize risk of damaging abdominal organs.

Another cause of collateral damage is electrosurgical injuries due to insulation failure. We routinely check insulation sheaths macroscopically for signs of wear; however, our institution lacks the facility to perform proper electrical conductivity tests on the instruments. Based on the literature, one in five RLIs has some kind of insulation defect, which tends to occur more often at the distal third of the sheath, near the working tip [27, 28]. Insulation problems have also been recorded with 3% of DLIs [27]. Electrosurgical injuries, especially in anatomic areas with delicate structures, may go undetected for months after surgery and are the cause of medicolegal actions [29].

Last but not least, increasing environmental concerns are not to be taken lightly [7, 13, 14, 30]. Laparoscopic instruments are manufactured using materials potentially hazardous to the environment. Additionally, plastic packaging results in large volume of unnecessary hospital waste. Surprisingly, we discovered that disposal cost of DLIs, based on their weight as waste, is practically negligible, compared to their purchase price [13]. However their impact on the environment is far greater than their impact on hospital budgets.

Cost studies of this type have a certain limitation. Our comparative analysis is based on specific retail prices for DLIs, obtained by the National Price Observatory. Suppliers of instruments may offer favorable pricing packages through individual agreements, when larger quantities are ordered [6]. Therefore our findings cannot be directly extrapolated to other institutions. However this study showed that RLIs have a clear financial advantage over the single-use platform. Choice of one over the other modality lies ultimately with the surgeon and should be guided not only by personal preference or habit, but also by cost-effectiveness [16, 31]. As Dr. Winter of the Institute for Clinical Outcomes Research and Education eloquently wrote, “*most of our modern disposable (and expensive) laparoscopic paraphernalia are non-essential luxuries. Reusable trocars and non-disposable instruments are often adequate, if not ideal*” [1].

The fact remains though that instrumentation cost is only a fraction of total hospital costs per patient [17]. For a sustainable health care management, a comprehensive approach to cost-effectiveness should also combine ambulatory protocols, targeted preoperative patient assessment, and standardized enhanced recovery pathways.

## 5. Conclusion

Reusable laparoscopic instruments substantially reduce the cost of laparoscopic surgery, without compromising safety of patients and medical personnel. Initial acquisition costs are quickly amortized and further depreciated with every use. Although they may be prone to more technical issues compared to disposable instruments, they should be considered an investment for surgical departments and handled with proper care and attention.

## Figures and Tables

**Table 1:** Laparoscopic operations between January 2012 and December 2013.

Operation	*n*
Cholecystectomy	454
Appendicectomy	42
Hernia repair	10
Nissen fundoplication	16
Colectomy	33
Adjustable gastric band	16
Vertical sleeve gastrectomy	20
Gastric plication	2
Heller's myotomy	1
Ventral rectopexy	1
Hepatic cyst unroofing	2
Exploratory/staging/biopsy	26

Total	623

**Table 2:** Replacement and repair requirements (incl. VAT 23%).

Part	Number of	Price per	Price (€)
	units	unit (€)	
Trocar valve 10–12 mm	50	8.65	432.35
Trocar valve 10–12 mm	56	76.20	4,267.12
with 5 mm adaptor			
Trocar valve 5 mm	79	12.77	1,008.62
Adaptor	36	70.11	2,523.96
Grasper tip repair	1	139.99	139.99
Scissors sharpening	1	Gratis	Gratis

		Total	8,372 Euros

**Table 3:** Hospital reimbursements by social security funds for common, uncomplicated laparoscopic procedures.

Operation	Hospital reimbursement (€)
Cholecystectomy	1,085
Appendicectomy	764
Hernia repair	868
Nissen fundoplication	1,649
Colon resection	2,519
Rectum resection	3,506
Obesity procedures	1,506
